# Determinants of health seeking behavior for chronic non-communicable diseases and related out-of-pocket expenditure: results from a cross-sectional survey in northern Bangladesh

**DOI:** 10.1186/s41043-019-0195-z

**Published:** 2019-12-23

**Authors:** Fatema Binte Rasul, Olivier Kalmus, Malabika Sarker, Hossain Ishrath Adib, Md Shahadath Hossain, Md Zabir Hasan, Stephan Brenner, Shaila Nazneen, Muhammed Nazmul Islam, Manuela De Allegri

**Affiliations:** 1 BRAC JPG School of Public Health, BRAC University, 68, Shahid Tajuddin Ahmed Sharani, Mohakhali, Dhaka, 1212 Bangladesh; 2 Heidelberg Institute of Global Health, Medical Faculty and University Hospital, Heidelberg University, Im Neuenheimer Feld 130.3, 69120 Heidelberg, Germany; 3 Working group for Translational Health Economics, Department for Conservative Dentistry, University Hospital Heidelberg, Heidelberg, 69120 Germany; 4 East West University, Dhaka, Bangladesh; 5 Practical Action, Bangladesh Country Programme, Dhaka, Bangladesh; 6 Binghamton University, 4400 Vestal Pkwy E, Binghamton, NY 13905 USA; 7 Department of International Health, Johns Hopkins Bloomberg School of Public Health, 615 N Wolfe St, Baltimore, MD 21205 USA

**Keywords:** Non-communicable diseases, Chronic illness, Health-seeking behavior, Out-of-pocket expenditure, Multinomial logistic regression

## Abstract

**Background:**

In spite of high prevalence rates, little is known about health seeking and related expenditure for chronic non-communicable diseases in low-income countries. We assessed relevant patterns of health seeking and related out-of-pocket expenditure in Bangladesh.

**Methods:**

We used data from a household survey of 2500 households conducted in 2013 in Rangpur district. We employed multinomial logistic regression to assess factors associated with health seeking choices (no care or self-care, semi-qualified professional care, and qualified professional care). We used descriptive statistics (5% trimmed mean and range, median) to assess related patterns of out-of-pocket expenditure (including only direct costs).

**Results:**

Eight hundred sixty-six (12.5%) out of 6958 individuals reported at least one chronic non-communicable disease. Of these 866 individuals, 139 (16%) sought no care or self-care, 364 (42%) sought semi-qualified care, and 363 (42%) sought qualified care. Multivariate analysis confirmed that the following factors increased the likelihood of seeking qualified care: a higher education, a major chronic non-communicable disease, a higher socio-economic status, a lower proportion of chronic household patients, and a shorter distance between a household and a sub-district public referral health facility. Seven hundred fifty-four (87 %) individuals reported out-of-pocket expenditure, with drugs absorbing the largest portion (85%) of total expenditure. On average, qualified care seekers encountered the highest out-of-pocket expenditure, followed by those who sought semi-qualified care and no care, or self-care.

**Conclusion:**

Our study reveals insufficiencies in health provision for chronic conditions, with more than half of all affected people still not seeking qualified care, and the majority still encountering considerable out-of-pocket expenditure. This calls for urgent measures to secure better access to care and financial protection.

## Background

Chronic non-communicable diseases (CNCDs) are controllable, although not curable conditions [1] that persist in individuals for a prolonged time, usually without any known transmitting agents [2]. The World Health Organization (WHO) highlighted that 68% of total worldwide deaths in 2012 were caused by CNCDs, and that three quarters of these deaths occurred in low- and middle-income countries (LMICs). Southeast Asia faced the highest increase in CNCD deaths [3].

Still, research on CNCDs in Southeast Asia is scarce, and it is mostly limited to establishing the prevalence of CNCDs and the associated risk factors [4, 5]. Little is known about how CNCD cases interact with the health system, with patients’ health-seeking choices, and with related out-of-pocket expenditure (OOPE) [6].

Likewise, Bangladesh has limited CNCD research, mostly focused on assessing the prevalence of selected CNCDs and their risk factors [7]. Although CNCDs account for 61% of the total disease burden in Bangladesh [8], few studies have explored related health seeking, and were concentrated in demographic surveillance sites in southern and central Bangladesh [9, 10]. Even fewer studies exist on OOPE for CNCDs, although evidence indicates that households affected by CNCD deaths are more likely to be impoverished [11].

The lack of information on health seeking choices and related expenditure makes it impossible to identify potential gaps in service provision and financial protection. In turn, an understanding of potential system failures in adequately addressing CNCDs is essential for designing policy reforms and programs that can effectively counteract the challenge posed by CNCDs, encourage movement towards universal health coverage, and consequently secure progress towards the Sustainable Development Goals (SDGs).

We aimed to fill this existing knowledge gap by exploring health-seeking behavior for CNCDs, its determinants, and related household OOPE in northern Bangladesh.

## Methods

### Study settings

Data for our study was collected in Rangpur district, located in northwestern Bangladesh. The district, which has a population of about 3 million people [12], experiences the highest poverty rate in the country, with 42% of all people living below the national poverty line [13]. Rangpur’s health system reflects medical pluralism in Bangladesh: there co-exist public, private for-profit, and private not-for-profit providers [14]. Although CNCD policies are in place [15, 16], their implementation has been slack [8]. In the public sector, tertiary facilities are prime providers for CNCDs [8, 17] (e.g., Rangpur Medical College hospital), whereas Upazilla Health Complexes (UHCs) offer basic services [17].

### Sampling

**Fig. 1:**
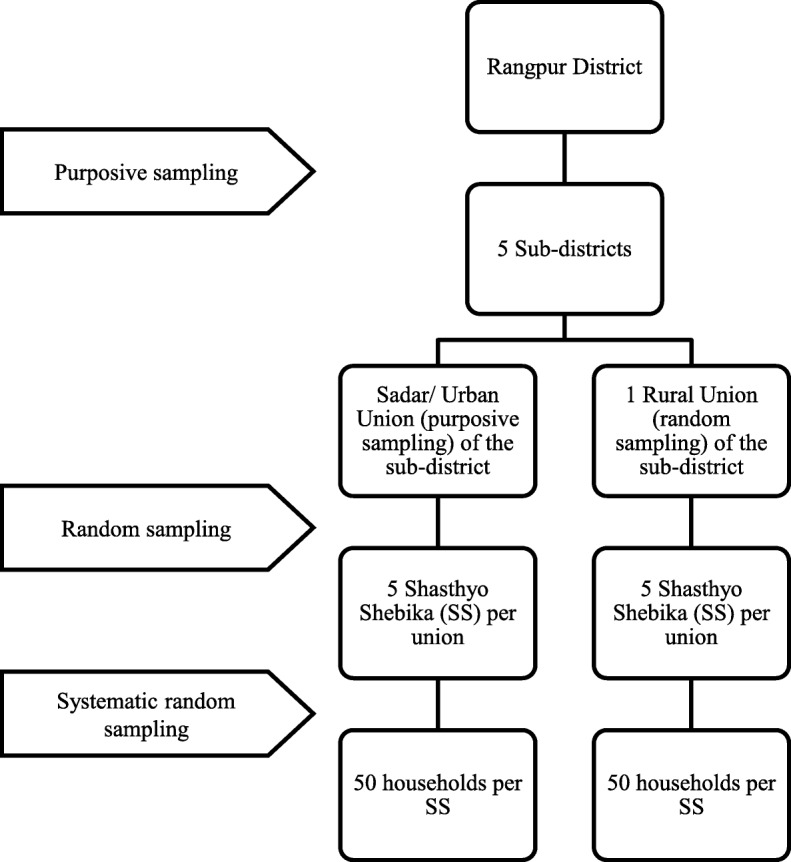
Flow chart showing sampling techniques used to select survey households

We used data from a household survey of 2500 households conducted in June–July 2013. It was a baseline or scoping survey for an upcoming health insurance scheme. The aim of the survey was to understand the practices and the differences among the sub-districts where the program was supposed to be implemented. The rationales for purposive sampling were driven by the needs of the upcoming health insurance program. Multi-stage cluster sampling techniques were applied to identify the households to be included in the survey. A mixture of random and purposive selection techniques were applied at each stage of sampling (Fig. 1). First, the survey purposely selected 5 out of 8 sub-districts (i.e., Upazilla): Rangpur Sadar, Badarganj, Mithapukur, Pirganj, and Pirgacha. These five sub-districts were selected purposively out of the 8 sub-districts as a programmatic decision, as a health coverage scheme was supposed to be rolled out in these 5 sub-districts. Second, within each sub-district, the Sadar union (i.e., main town of the sub-district) was purposely selected, and another union from the remaining unions (5–17 unions per sub-district) was randomly selected. The purposive selection of 5 main towns of 5 sub-districts was also a programmatic decision, taken with the intention to see if the circumstances of a sub-district’s Sadar Union (i.e., main town) differs from the rest of the unions. In each sub-district, there is one union that is considered the main town (called Sadar Union), which is either an urban or peri-urban area, and the remaining unions are considered rural areas. Third, in each union, we randomly selected 5 out of 50–55 BRAC Shasthyo Shebika^1^ (SS), i.e., BRAC community health volunteers. Finally, we used systematic random sampling to select 50 households among each SS’s target population (150–200 households).

### Data collection

Trained enumerators administered a structured questionnaire to sampled households. Household heads and their spouses responded on behalf of all individuals living in a household. The questionnaire gathered information on the household’s socio-demographic and economic profile, self-reported illnesses (both acute and chronic conditions), and related health-seeking behavior, health expenditure, and participation in microfinance institutions. Enumerators also recorded the household’s Global Position System (GPS) location.

The survey defined chronic conditions as any condition that had lasted 3 months or more. The questionnaire explicitly probed for name and symptoms of chronic conditions expected to be included in a prospective insurance benefit package: hypertension, diabetes, asthma or chronic obstructive pulmonary disease (COPD), physical disability, joint pain or arthritis, cancer, chronic communicable conditions (tuberculosis, leprosy, kala-azar, and polio), and other chronic diseases. If respondents reported conditions beyond probed ones, they categorized it as “other conditions.” Because our focus was on CNCDs, we excluded chronic communicable conditions.

The Ethical Review Committee of the BRAC JPG School of Public Health, BRAC University reviewed and approved the study protocol shortly before data collection began in 2013. Interviewers obtained written informed consent from all respondents before interview.

### Variables

We defined the primary outcome variable, health-seeking behavior, as the type of care sought by individuals reporting at least one CNCD in the past 30 days. The survey gathered self-reported information of health-seeking behavior and related expenditure for the past 30 days rather than for a longer 12-month period, as shorter recall periods have minimal recall bias and are more accurate [18, 19]. Shorter recall periods are more appropriate when capturing micro level data than are longer recall periods [20]. Moreover, our study has followed a similar study done recently in Malawi (another low-resource setting like Bangladesh), where a 30-day recall period had been used to collect self-reported health-seeking information and expenditure related to chronic non-communicable diseases [21].

We categorized care seeking as: *no care* or *self-care*, *semi-qualified professional care,* and *qualified professional care*. From a conceptual viewpoint, this classification reflects real-life alternatives available in the pluralistic Bangladeshi context.

We defined instances as *no care* when a person did nothing to treat the reported condition and as *self-care* when a person engaged in treatment without the recommendation of a health provider, but instead followed their own advice or that of a family or friend [22]. We merged *self-care* and *no care* into one category owing to the low response rate, but applied the likelihood-ratio test in order to test beforehand for the feasibility of combining these two alternatives [23]. We defined instances as *semi-qualified professional care* when a person sought care from any allopathic or traditional provider with some degree of training and experience in primary care, but no specific expertise in CNCDs (e.g., medical assistants, village doctors, community health workers, drugstore keepers, and traditional healers) [9, 18, 22]. We defined instances as *qualified professional care* when a person sought care from registered and trained physicians (i.e., MBBS doctors) [9, 22].

We defined the secondary outcome variable as *total OOPE,* incurred while seeking CNCD care during the prior 30 days, irrespective of sought care type. Our estimate included self-reported expenses for consultation, medications, diagnostics, transportation, and other related direct costs (e.g., informal pay, and accommodation). We could not analyze the single cost components of total OOPE except for medication expenses, owing to respondents’ difficulty in recalling them. We did not collect information on indirect costs.

Our selection of explanatory variables was guided by Andersen’s model of health-seeking behavior [24]. We have listed all explanatory variables with a hypothesized association with primary outcome in Table 1. Most of them are self-explanatory and reflect standard measurement practice in analyses pertaining to health-seeking behavior [21, 22].

**Table 1:** Variables, their measurements, and hypotheses

Variables and their measurement	Hypothesized direction of explanatory variables’ influence on primary outcome^1^	
	No/self-care vs. qualified care	Semi-qualified care vs. qualified care
Primary outcome variable: type of health seeking		
1 = No care/self-care	NA	NA
2 = Semi-qualified professional care		
3 = Qualified professional care		
Secondary outcome variable: total out-of-pocket expenditure incurred to seek CNCD care in prior 30 days (Continuous variable)		
**Explanatory variables**		
*Individual characteristics*		
Age: Continuous variable	+/-	+/-
Duration of illness (months): Continuous variable	-	-
Sex: 0 = Male 1 = Female	+/-	+/-
Education: 0 = No schooling 1 = Primary level and above	-	-
Marital status: 0 = Currently not married 1 = Currently married	+/-	+/-
Occupational status: 0 = Non-income generating 1 = Income generating	-	-
Being household head: 0 = No 1 = Yes	-	-
Comorbidity: 0 = No comorbidity 1 = Comorbidity	-	-
Category of CNCDs: 0 = Minor CNCDs, 1 = Major CNCDs	-	-
*Household characteristics*		
Household size (no. of members): continuous variable	+	+
Socio-economic status/ asset quintiles 1 = 1st quintile (poorest) 2 = 2nd quintile 3 = 3rd quintile 4 = 4th quintile 5 = 5th quintile (least poor)	-	-
Proportion of household members with CNCD: continuous variable	+	+
MFI involvement of household head and/or spouse: 0 = Not involved with MFI 1 = Involved with MFI	-	-
Presence of acute illness in the household: 0 = No, 1 = Yes	+	+
*Contextual characteristics*		
Distance between household and sub-district’s public health care facility (Upazilla Health Complex/Medical college hospital): continuous variable	+	+
Rural or urban residence: 0 = Rural, 1 = Urban	-	-
Sub-district of residence : 1 = Rangpur Sadar 2 = Mithapukur 3 = Badarganj 4 = Pirganj 5 = Pirgacha	+/-	+/-

*CNCD*, chronic non-communicable diseases; *MFI*, Micro-Finance Institute

^1^Direction of influence: positive association (+), negative association (-), not sure of direction of association (+/-)

To explore the effect of different CNCDs on care seeking while accounting for small numbers, we re-classified CNCDs into two groups: major CNCDs and minor CNCDs. Corresponding to disease burden estimates in Bangladesh and in South-Asia [15, 25, 26], we categorized hypertension, asthma/COPD, diabetes, and cancer as “major CNCDs” because they underlie the four leading causes of CNCD deaths: cardiovascular diseases, respiratory diseases, cancer, and diabetes [3, 25]. We categorized the remaining conditions as “minor CNCDs” (chronic joint pain or arthritis, physical disability, chronic gastro-intestinal conditions, and other CNCDs), as they impose a lower disease burden [25, 26] and are less central in the local discourse on CNCDs [15, 25, 26].

We included being a household head as an explanatory variable because we expected intra-household allocation of resources to be in his/her favor, as shown by a prior study in Malawi [21]. We included microfinance participation (by the household head and/or his/her spouse) because we postulated that it may facilitate access to resources and therefore to care [27]. We included the presence of an acute illness episode in the household in the prior 30 days because we assumed a reduced ability to seek CNCD care owing to competing health needs within a context of limited household resources [28]. Socio-economic status was measured by constructing asset quintiles, a relative score obtained by assembling household belongings, calculated by principal component analysis (PCA) [29]. The following household assets were factored in: house ownership, house infrastructure (roof material, type of toilet, number of rooms), primary source of drinking water, cooking fuel, light source (electricity, kerosene oil, or candles), land ownership, durable assets ownership (bicycles, tri-cycled van or rickshaw, motor-bikes, cars, other motorized vehicles, tube-well^2^, pond^3^, sewing machine, television, computer, and gold), and animal ownership (cows, goats, hens, ducks, pigeons). To generate cutoff points, we simply used quintiles; hence, after ordering the index, we defined a quintile in relation to the 20% of the population below a given index value.

To test the effect of distance on access to care, we included a measure of distance by computing the shortest ellipsoidal length between the household GPS coordinates and the sub-district public referral health facility. We used the sub-districts’ public referral facilities in this computation because they are expected to provide CNCD services [8, 17].

### Analytical Approach

We conducted our analysis using STATA IC 13. We considered all results with *P* values less than 0.05 as statistically significant. We used univariate and bivariate descriptive statistics (analysis of variance-ANOVA, chi-square, or Fisher’s exact test) to explore the distribution of the variables and to identify associations with health-seeking behavior.

We used multinomial logistic regression (MNL) to confirm the associations identified in the bivariate analysis, between explanatory variables and health-seeking choices. We used MNL because our primary outcome variable included three answer categories (no care or self-care, semi-qualified professional care, and qualified professional care). The equation is [23] as follows:

$$ \Pr \kern0.28em \left(\mathrm{y}=\mathrm{m}|\kern0ex \mathrm{x}\right)=\frac{\exp \left({\mathrm{x}\upbeta}_{\mathrm{m}\mid 3}\right)}{\sum \limits_{\mathrm{j}=1}^{\mathrm{J}}\exp \left({\mathrm{x}\upbeta}_{\mathrm{j}\mid 3}\right)},\mathrm{for}\kern0.28em m=1,2\kern0.28em or\kern0.28em 3 $$

Here “*m* = 1” is seeking no care or self-care, “*m* = 2” is seeking semi-qualified professional care and “*m* = 3” is seeking qualified professional care. We set care by qualified professionals as the base category because they are considered the highest-level health providers in Bangladesh [9, 22, 30] and are expected to provide adequate CNCD care. By setting them as a reference category, we effectively measured which individual, household, and contextual characteristics prevented people from accessing proper care.

We used a step-up approach to build our MNL model [31]. We started by running the MNL model with intercept only. We progressively added one explanatory variable each time to the model, privileging variables that had shown a significant association in bivariate analysis. After adding a new variable, we tested the model against the prior model using the likelihood ratio test. If the prior model was nested in a later model including an additional variable, then we kept the added variable. If not, we dropped the added variable. We repeated this process until we identified the final model. This approach explains why the final model contains fewer variables than those we had originally considered. We used the Hausman test and Small-Hsiao test to test the model assumption of Independence of Irrelevant Alternatives (IIA) [23].

We analyzed OOPE and its components in Bangladeshi Taka (BDT) (1USD~78 BDT as of June–July, 2013, when data was collected). We used univariate descriptive statistics (5% trimmed mean and range (minimum-maximum), and median) to explore expenditure patterns and their distribution across health-seeking choices, individual, household, and contextual characteristics.

## Results

We collected information on a total of 10,367 individuals, of which 6958 people were aged 15 years or above, and were therefore included in our analysis on CNCDs. Among those, 866 (12.5%) reported a total of 925 CNCDs. The characteristics of the entire sample and the respondents who had at least one CNCD are given in Table 2.

**Table 2:** Socio-demographic and CNCD-related characteristics of entire sample and CNCD respondents

Variable	Entire sample		CNCD sample^1^	
	*N* = 6958		*N* = 866	
* Continuous variables*	**Mean**	**SD**	**Mean**	**SD**
Age (years)	35.99	15.21	43.83	16.14
Distance to the referral public health facility of the sub-district (km)	5.54	3.82	4.91	3.63
Household size (number of members)	4.64	1.78	4.32	1.71
Duration of illness (months)	NA	NA	43.77	60.76
Proportion of household members with CNCD	NA	NA	0.41	0.24
* Categorical variables*	***N***	**%**	***N***	**%**
Sex:				
Male	3518	50.56	401	46.3
Female	3440	49.44	465	53.7
Education**:**				
No schooling	2751	39.55	435	50.23
Primary level education and above	4205	60.45	431	49.77
Marital status:				
Currently not married	1276	18.34	75	8.66
Currently married	5682	81.66	791	91.34
Occupational status:				
Non-income generating	3749	53.88	486	56.12
Income generating	3209	46.12	380	43.88
Presence of acute illness patient in household:				
No	2471	35.51	204	23.56
Yes	4487	64.49	662	76.44
Category of CNCDs:				
Minor CNCDs	NA	NA	555	64.09
Major CNCDs	NA	NA	311	35.91
Asset Quintile/socio-economic status:				
1st quintile (poorest)	1095	15.74	155	17.9
2nd quintile	1256	18.05	163	18.82
3rd quintile	1351	19.42	150	17.32
4th quintile	1479	21.26	183	21.13
5th quintile (least poor)	1777	25.54	215	24.83
Being household head:				
No	4466	64.19	492	56.81
Yes	2492	35.81	374	43.19
Microfinance involvement of household head and/or spouse:				
Not involved	3962	56.94	410	47.34
Involved	2996	43.06	456	52.66
Rural or urban residence:				
Rural	3372	48.46	364	42.03
Urban	3586	51.54	502	57.97
Sub-district of residence:				
Rangpur Sadar	1341	19.27	83	9.58
Mithapukur	1394	20.03	473	54.62
Badarganj	1473	21.17	49	5.66
Pirganj	1298	18.65	109	12.59
Pirgacha	1452	20.87	152	17.55

*CNCDs*, chronic non-communicable diseases; *NA*, not applicable; *SD*, standard deviation; *km*, kilometer

^1^Respondents reported to have at least one chronic non-communicable disease

The three most commonly reported CNCDs were chronic joint pain/arthritis (*n* = 162), asthma/COPD (*n* = 151), and hypertension (*n* = 105) (Table 3). Among individuals with at least one CNCD, 139 (16%) sought no care or self-care, 364 (42%) sought semi-qualified care, and 363 (42%) sought qualified care (Table 4).

**Table 3:** Reported cases and proportions per CNCD category

Type of CNCD	Name of CNCD	*N*	Percentage (%)
Major CNCDs (*n* = 311)	Hypertension	105	11.35
	Diabetes	64	6.919
	Asthma/COPD	151	16.32
	Cancer	10	1.08
Minor CNCDs (*n* = 555)	Physical disability	23	2.49
	Joint pain/arthritis	162	17.51
	Chronic gastro-intestinal conditions	16	1.73
	Other chronic diseases	394	42.59
	Total episodes	925	100

*CNCD*, chronic non-communicable disease; *N*, number; *COPD*, chronic obstructive pulmonary disease

This table shows the total conditions and their proportions as reported by 866 respondents. The conditions of 866 respondents add up to 925 because some people reported more than one condition

**Table 4:** Bivariate analysis between type of health care-seeking behavior and explanatory variables, (*N* = 866)

Variable and its measurement	No care or self-care (*N* = 139)	Semi-qualified professional care *(N* = 364)	Qualified professional care *(N* = 363)	Test statistics and *P* value
*Individual characteristics*				
Age (years), mean (SD)	44.0 (16.5)	42.6 (16.1)	45.0 (16.1)	*F* (2, 863) = 1.92, *P* = 0.15^1^
Duration of illness (months), mean (SD)	53.6 (60.1)	44.7 (65.6)	39.1 (55.4)	*F* (2, 863) = 2.95, *P* = 0.05^1^
Sex, *n* (%)				
Male	63 (45.3)	174 (47.8)	164 (45.2)	*X*^2^ = 0.57, df = 2, *P* = 0.75^2^
Female	76 (54.7)	190 (52.2)	199 (54.8)	
Education, *n* (%)				
No schooling	83 (59.7)	189 (51.9)	163 (44.9)	*X*^2^ = 9.54, df = 2, *P* = 0.008^2^
Primary level and above	56 (40.3)	175 (48.1)	200 (55.1)	
Marital status, *n* (%)				
Currently not married	14 (10.1)	34 (9.3)	27 (7.4)	*X*^2^ = 1.25, df = 2, *P* = 0.54^2^
Currently married	125 (89.9)	330 (90.7)	336 (92.6)	
Occupational status, *n* (%)				
Non-income generating	77 (55.4)	202 (55.5)	207 (57.0)	*X*^2^ = 0.21, df = 2, *P* = 0.90^2^
Income generating	62 (44.6)	162 (44.5)	156 (43.0)	
Being household head, *n* (%)				
No	77 (55.4)	202 (55.5)	213 (58.7)	*X*^2^ = 0.89, df = 2, *P* = 0.64^2^
Yes	62 (44.6)	162 (44.5)	150 (41.3)	
Comorbidity, *n* (%)				
No comorbidity	122 (87.8)	342 (94.0)	345 (95.0)	*X*^2^ = 8.94, df = 2, *P* = 0.01^2^
Comorbidity	17 (12.2)	22 (6.0)	18 (5.0)	
Category of CNCDs, *n* (%)				
Minor CNCDs	103 (74.1)	267 (73.4)	185 (51.0)	*X*^2^ = 46.79, df = 2, *P* < 0.001^2^
Major CNCDs	36 (25.9)	97 (26.7)	178 (49.0)	
*Household characteristics*				
Household size (members), mean (SD)	4.25 (1.68)	4.15 (1.63)	4.52 (1.78)	*F* (2, 863) = 4.29, *P* = 0.01^1^
Proportion of household members with CNCD, mean (SD)	0.46 (0.25)	0.45 (0.25)	0.34 (0.21)	*F* (2, 863) = 22.89, *P* < 0.001^1^
Asset Quintile, *n* (%)				
1st quintile (Poorest)	31 (22.3)	88 (24.2)	36 (9.9)	*X*^2^ = 35.35, df = 8, *P* < 0.001^1^
2nd quintile	24 (17.3)	69 (19.0)	70 (19.3)	
3rd quintile	22 (15.8)	62 (17.0)	66 (18.2)	
4th quintile	32 (23.0)	75 (20.6)	76 (20.9)	
5th quintile	30 (21.6)	70 (19.2)	115 (31.7)	
Microfinance involvement of household head and/or spouse, *n* (%)				
No	57 (41.0)	173 (47.5)	180 (49.6)	*X*^2^ = 2.98, df = 2, *P* = 0.23^2^
Yes	82 (59.0)	191 (52.5)	183 (50.4)	
Presence of acute illness in the household, *n* (%)				
No	34 (24.5)	60 (16.5)	110 (30.3)	*X*^2^ = 19.35, df = 2, *P* < 0.001^2^
Yes	105 (75.5)	304 (83.5)	253 (69.7)	
*Contextual characteristics*				
Distance to the referral public health facility of the sub-district (km), mean (SD)	4.82 (3.69)	5.25 (3.64)	4.59 (3.57)	*F* (2, 863) = 3.03, *P* = 0.05^1^
Rural or urban residence, *n* (%)				
Rural	54 (38.9)	165 (45.3)	145 (39.9)	*X*^2^ = 2.85, df = 2, *P* = 0.24^2^
Urban	85 (61.2)	199 (54.7)	218 (60.1)	
Sub-district of residence, *n* (%)				
Rangpur Sadar	16 (11.5)	25 (6.9)	42 (11.6)	*X*^2^ = 85.11, df = 8, *P* < 0.001^2^
Mithapukur	98 (70.5)	232 (63.7)	143 (39.4)	
Badarganj	4 (2.9)	8 (2.2)	37 (10.2)	
Pirganj	5 (3.6)	33 (9.1)	71 (19.6)	
Pirgacha	16 (11.5)	66 (18.1)	70 (19.3)	

*CNCDs*, chronic non-communicable diseases; *F*, F statistic; *X*^2^, chi-square value; *df*, degree of freedom; *SD*, standard deviation; *km*, kilometer

^1^Test statistics and *P* values based on ANOVA for continuous variables

^2^Test statistics and *P* values based on chi^2^ tests (or Fisher exact tests) for categorical variables

Table 4 reports the bivariate analysis results between their health seeking choices and explanatory variables. We found a positive association between seeking no or self-care and longer illness duration (*P* = 0.05), increasing proportion of household CNCD members (*P* < 0.001), and Mithapukur residents (*P* < 0.001). Respondents with primary education or more (*P* = 0.01), major CNCDs (*P* < 0.001), and from 2nd and 3rd asset quintiles (*P* < 0.001) were less likely to seek no or self-care.

Longer illness duration (*P* = 0.05), increasing proportion of household CNCD patients (*P* < 0.001), presence of acute illness in a household (*P* < 0.001), longer distance from the sub-district’s public referral health facility (*P* = 0.05), and Mithapukur and Pirgacha residents (*P* < 0.001) were more likely to seek semi-qualified care. Major CNCD patients (*P* < 0.001) and respondents from higher asset quintiles (*P* < 0.001) were less likely to seek semi-qualified care.

Table 5 reports the results of MNL and model specifications. MNL analysis confirmed that respondents with primary education or more (*β* = − 0.624, *P* = 0.007), with major CNCDs (*β* = − 0.523, *P* = 0.03), and from 2nd (*β* = − 0.794, *P* = 0.03), or 3rd asset quintiles (*β* = − 0.841, *P* = 0.02) were less likely to seek no or self-care, compared to qualified care. It also confirmed that people from households with a higher proportion of CNCD patients (*β* = 1.561, *P* = 0.001), and from Mithapukur (*β* = 1.040, *P* = 0.01), were more likely to seek no or self-care than qualified care. However, MNL could not confirm associations between no or self-treatment and illness duration.

**Table 5:** Health-seeking behavior for CNCDs: estimated coefficients in multinomial logistic regression model

Type of health seeking^1^	No care or self-care vs. qualified professional care			Semi-qualified pr. care vs. qualified professional care		
	*β* coefficient	95% CI	*P* value	*β* coefficient	95% CI	*P* value
Intercept	− 1.878	− 3.92, 0.16	0.07	− 2.791	− 4.39, − 1.19	0.001
*Individual characteristics*						
Primary level education and above (reference group: no schooling)	− 0.624	− 1.07, − 0.17	0.007	− 0.187	− 0.53, 0.15	0.28
Have major CNCD (reference group: have minor CNCD)	− 0.523	− 1.01, − 0.04	0.03	− 0.665	− 1.02, − 0.31	< 0.001
*Household characteristics*						
Proportion of CNCD patients in household	1.561	0.64, 2.49	0.001	1.522	0.78, 2.27	< 0.001
Asset quintile (reference group: 1st quintile)						
2nd quintile	− 0.794	− 1.50, − 0.09	0.03	− 0.893	− 1.44, − 0.35	0.001
3rd quintile	− 0.841	− 1.57, − 0.12	0.02	− 0.872	− 1.43, − 0.31	0.002
4th quintile	− 0.498	− 1.19, 0.19	0.16	− 0.783	− 1.33, − 0.23	0.005
5th quintile (least poor)	− 0.627	− 1.33, 0.08	0.08	− 0.987	− 1.54, − 0.43	< 0.001
Presence of acute illness in household (reference group: no acute illness in household)	− 0.468	− 1.02, 0.08	0.10	0.308	− 0.12, 0.74	0.16
*Contextual characteristics*						
Distance to sub-district’s public referral health facility	0.140	− 0.03, 0.31	0.11	0.232	0.10, 0.36	< 0.001
Type of residence (reference group: rural)						
Urban	0.951	− 0.23, 2.13	0.11	1.297	0.39, 2.21	0.005
Sub-district of residence (reference group: Rangpur Sadar)						
Mithapukur	1.040	0.25, 1.83	0.01	1.458	0.80, 2.12	< 0.001
Badarganj	− 0.393	− 1.81, 1.03	0.59	0.623	− 0.48, 1.73	0.27
Pirganj	− 1.166	− 2.35, 0.02	0.05	0.637	− 0.13, 1.40	0.10
Pirgacha	0.112	− 0.85, 1.07	0.82	1.457	0.71, 2.20	< 0.001
*Multinomial logistic regression model specifications:*						
Pseudo *R*^2^ = 0.1028	*X*^2^ (28) = 182.04, *P > X*^2^ = 0.0000					
Hausman tests of IIA assumption	*X*^2^ (15) = 14.71 (omitted semi-qualified care), *P > X*^2^ = 0.473, *X*^2^ (15) = 10.37 (omitted no/self-care), *P > X*^2^ = 0.796					
Small-Hsiao tests of IIA assumption	*X*^2^ (15) = 12.35 (omitted semi-qualified care), *P > X*^2^ = 0.652, *X*^2^ (15) = 9.27 (omitted no/self-care), *P > X*^2^ = 0.863					

C*NCDs*, chronic non-communicable diseases; *pr*., professional; *CI*, confidence interval; *IIA*, independence of irrelevant alternatives

^1^We considered qualified professional care seekers as reference category for multinomial logistic regression

MNL analysis affirmed that households with a higher proportion of CNCD patients (*β* = 1.522, *P* < 0.001), a longer distance from the sub-district’s public referral health facility (*β* = 0.232, *P* < 0.001), urban respondents (*β* = 1.297, *P* = 0.01), and Mithapukur (*β* = 1.458, *P* < 0.001), or Pirgacha residents (*β* = 1.457, *P* < 0.001) were more likely to seek semi-qualified care, compared to qualified professional care, and respondents with major CNCDs (*β* = − 0.665, *P* < 0.001), and from 2nd (β = − 0.893, *P* = 0.001), 3rd (β = − 0.872, *P* = 0.002), 4th (*β* = − 0.783, *P* = 0.005), or 5th (*β* = − 0.987, *P* < 0.001) asset quintiles were less likely to seek semi-qualified care than qualified care. The MNL did not confirm associations between illness duration, the presence of an acute illness in a household, and the seeking of semi-qualified care.

Out of 866 respondents with a CNCD, 754 (87%) reported regarding OOPE in the prior 30 days, and 85% of total OOPE consisted of drug expenditure. Table 6 shows the distribution of total OOPE and drug expenses across variables. People who sought qualified professional care, people suffering from a major CNCD, the elderly (60 years old and above), and the least poor incurred the highest OOPE. Important differences were observed across sub-districts, with Mithapukur residents facing the lowest OOPE and Pirgacha residents facing the highest.

**Table 6:** Distribution of total out-of-pocket expenditure (OOPE) and expenditure for drugs (in BDT)

Variable and sub-category		Total direct OOPE^1^ (*N* = 754)^2^			Expenditure on drugs^3^ (*N* = 728) ^4^		
		5% trimmed		Median^7^	5% trimmed		Median^10^
		Mean^5^	Range^6^ (min-max)		Mean^8^	Range^9^ (min-max)	
Type of care sought	No care or self-care	466.2	(10–4050)	200	372.8	(10–3000)	175
	Semi-qualified care	765.9	(30–5000)	350	535.1	(30–3000)	300
	Qualified care	3224.6	(200–18000)	2000	1811.3	(50–11000)	1000
Age	Productive-age group (15 < 60 years)	1647.5	(50–10500)	800	961.6	(40–6000)	500
	Elderly (≥ 60 years)	2495.4	(30–23300)	1000	1727.9	(50–20000)	500
Sex	Male	1753.3	(50–12000)	750	1018.8	(50- 5000)	500
	Female	1787.3	(40–13000)	825	1092.0	(40–8000)	500
Education	No schooling	1538.3	(30- 10880)	630	1062.9	(30–8900)	500
	Primary education and above	1969.6	(60–13000)	975	1066.5	(50- 6000)	500
Status of occupation	Not income generating	2012.6	(50–14450)	885	1192.0	(50–8000)	500
	Income generating	1525.4	(50- 8000)	700	931.0	(40–5000)	500
Being household head	No	1847.1	(50–13005)	910	1122.5	(45–8000)	500
	Yes	1652.3	(50–10500)	700	993.3	(40–5000)	500
Type of CNCD	Major CNCD	2313.4	(100–2000)	1250	1343.4	(100–7000)	600
	Minor CNCD	1453.3	(40–13000)	550	888.1	(30- 8000)	400
Asset quintile	1st quintile (poorest)	1722.9	(30–15000)	550	1000.7	(30–10000)	500
	2nd quintile	1318.5	(50–7200)	700	893.4	(50–5000)	500
	3rd quintile	1749.6	(30- 9000)	850	1075.2	(30–6000)	500
	4th quintile	1373.4	(40–8000)	700	861.6	(35–5000)	500
	5th quintile (least poor)	2690.3	(90–16000)	1500	1494.0	(60–10000)	600
Type of residence	Rural	2080.9	(75- 13000)	1000	1218.5	(50–8000)	600
	Urban	1542.2	(30- 12000)	700	945.2	(40–7000)	500
Sub-district of residence	Rangpur Sadar	2211.9	(50–15000)	1160	1465.2	(50–8900)	775
	Mithapukur	1034.3	(30–8000)	400	626.5	(30–4000)	300
	Badarganj	3537.7	(400–2000)	2350	1633.0	(200–10000)	900
	Pirganj	1941.2	(120- 10120)	1111.5	1206.9	(100–5000)	500
	Pirgacha	3539.5	(25–20000)	2100	2311.4	(100–15000)	1200

*OOPE*, out-of-pocket expenditure; *CNCD*, chronic non-communicable disease; *BDT*, Bangladeshi Taka

^1^Total OOPE consists of expenditure for consultation fee, drugs, diagnostics, informal pay and transport cost. The expenditure is shown in Bangladeshi taka (BDT). Exchange rate of data collection period (June–July, 2013), 1 USD~78 BDT

^2^Not all CNCD respondents incurred expenditure or reported on it. We found 754 respondents out of 866 who reported about OOPE

^3^We show expenditure for drugs besides total OOPE, because it constituted the largest component (85%) of total OOPE. The expenditure is shown in Bangladeshi taka (BDT). Exchange rate of data collection period (June–July, 2013), 1 USD~78 BDT

^4^Most respondents reported a lump-sum OOPE and had difficulty recalling cost breakdowns. This is the reason we have fewer observations for expenditure on drugs compared to observations of total OOPE

^5^We observed skewed distribution of OOPE. Therefore, we reported a 5% trimmed mean

^6^We observed skewed distribution of OOPE. Therefore, we reported a 5% trimmed range (minimum-maximum)

^7^Median of all OOPE observations (754 observations)

^8^We observed skewed distribution of expenditure on medications. Therefore, we reported a 5% trimmed mean

^9^We observed skewed distribution of drug costs. Therefore, we reported a 5% trimmed range (minimum-maximum)

^10^Median of all observations that reported on drug expenditure (728 observations)

## Discussion

Our work makes an important contribution to the limited pool of literature addressing health-seeking behavior for CNCDs and related OOPE, being one of the very few relevant studies in Southeast Asia, particularly in Bangladesh. Moreover, our study distinguishes itself from prior studies [9, 10] because, being based on population-based data, it addresses a wider spectrum of CNCDs experienced directly by the respondents.

One out of every eight respondents reported at least one CNCD, with the most commonly reported conditions being joint pain/arthritis, asthma/COPD, and hypertension. Despite our intention not to derive any epidemiological estimate of disease prevalence, our findings are consistent with prior evidence from INDEPTH surveillance sites in Asia, including Bangladesh [4].

Among those who reported at least one CNCD, an impressive 84% sought some sort of care. Contrary to previous findings [9, 10], our study showed an equal split between the seeking of qualified (42%) and of semi-qualified (42%) care. Furthermore, our findings indicated that irrespective of provider choice, individuals faced considerable OOPE, mostly owing to medication costs. Still, individuals who sought qualified care spent substantially higher amounts, suggesting a higher potential for catastrophic spending and impoverishment in this group. Substantial OOPE indicates that national policies stipulating CNCD prevention and control [15, 16] are failing to translate into a corresponding reality [8, 32], pushing people to purchase services and drugs at private providers [17]. This policy-implementation gap probably explains why such a large proportion of respondents bypassed the formal system and sought semi-qualified care. This obviously raises fundamental questions about the adequacy and quality of the care received [33], with important implications for disease control.

Among the individual characteristics affecting service provider choice, gender and education stand most prominent, and age to some extent. We found that lower education limits access to qualified care. This depicts the role of cultural capital (beyond socio-economic status) in shaping health seeking decisions [9] and urgently calls for interventions specifically reaching out to people with low educational levels. In contrast to prior literature on health seeking [9, 34], we found no evidence of a gender bias in health-seeking behavior and related expenditure. This appears surprising and calls for further qualitative inquiry to understand whether unexplored factors specific to CNCDs may mediate a different relation between gender and health-seeking behavior. Since our model could not be adjusted to control for illness reporting bias, we cannot exclude that in reality, gender plays a role already at the level of illness reporting, before the individual is even confronted with decision-making on seeking care [35]. Deeper understanding is essential to inform future policies and interventions. In line with prior studies from Bangladesh [34], we found higher health expenditure (CNCD-related expenditure in this study) among the elderly (60 years old and above). This finding is not surprising, since, consistent with economic theory [36], one would expect the need for medication to increase with age as health deteriorates. However, the finding is worrisome since it points at the potential for the elderly, i.e., those most in need, to forgo care owing to the fear of incurring high costs. Further qualitative inquiry is needed to clarify the role of age in mediating decisions concerning health-care seeking and specifically health spending.

The fact that individuals suffering from major CNCDs were more likely to seek qualified care and incur higher expenditure is likely a reflection of existing health system structures and policies [15], and emphasizes these conditions as the ones incurring the highest burden in the country. Additionally, given the importance that major CNCDs receive in the national discourse on CNCDs [15, 32], it is likely that cases of individuals affected by major CNCDs generate a higher degree of perceived severity [21] than do cases of minor CNCDs. As our study did not include a measure of perceived severity, qualitative inquiry is required to explore this issue further.

Our findings echo prior results from low-resource settings, showing that the chances of seeking qualified care decrease as the proportion of household members suffering from CNCDs increases [21]. This is likely the consequence of decisions on intra-household resource allocation, with heavily affected households having to ration health spending to avoid asset depletion [21, 28]. In line with prior evidence from Bangladesh [9, 22], appraising these findings jointly with findings indicating a higher propensity to use qualified care among the least poor, and with findings suggesting the regressive nature of OOPE, points at the existing gaps in population coverage and financial protection. In turn, recognition of these gaps calls for the urgent introduction of measures to ensure equitable access and financial protection for affected households.

Our study also identified an increasing distance to the sub-district public referral facility, as well as urban residence as factors affecting the probability to seek qualified care. While the relation between formal service use and distance is self-explanatory and has been widely documented, the relationship between urban residence and health choices appears surprising and requires further investigation. Similarly, the differences observed across sub-districts can only be explained and understood through further qualitative inquiry. It is plausible to assume that the difference observed across rural and urban contexts and across sub-districts is the result of specific features in the local health system organization, which could not be captured in our survey.

## Conclusions

In a context where primary government facilities do not offer CNCD care [8], care seeking for CNCD remains problematic. Our study clearly identifies some key challenges and, in doing so, points to the urgent need to fill the policy-implementation gap.

## Data Availability

The data used for this study are not publicly available. Data can be requested from BRAC JPG School of Public Health, BRAC University, but restrictions may apply. Requests should be directed to Prof. Malabika Sarker, Professor, Director Research and Associate Dean, BRAC JPG School of Public Health, BRAC University.

## Abbreviations

ANOVA: Analysis of variance
CNCDs: Chronic non-communicable diseases
COPD: Chronic obstructive pulmonary disease
GPS: Global position system
INDEPTH: International Network for the Demographic Evaluation of Populations and Their Health
LMICs: Low- and middle-income countries
MBBS: Bachelor of Medicine and Surgery
MNL: Multinomial logistic regression
OOPE: Out-of-pocket expenditure
PCA: Principal component analysis
SDGs: Sustainable development goals
SS: Shasthyo Shebika
UHCs: Upazilla health complexes
WHO: World Health Organization

## Footnotes

1 Shasthyo Shebika (SS) are a set of female community health volunteers who are trained by BRAC to provide essential health care services in the communities. They are volunteers, not workers of BRAC; they are not paid by BRAC but earn money by selling basic medications and services. At present, numbers of SS have scaled up to nearly 100,000 from around 1,000 in year 1990, operating in all districts of Bangladesh.

2 Tube-wells are a source of drinking water. But not all households that have tube-wells drink water from them.

3 As the population’s health consciousness increases, ponds are less likely to be a source of drinking water: they are more of a household cleaning water source.

## References

[1] O’Halloran J, Miller GC, Britt H. Defining chronic conditions for primary care with ICPC-2. Fam Pract [Internet]. 2004 [cited 2016 Jan 19];21:381–6. Available from: http://www.fampra.oupjournals.org/cgi/doi/10.1093/fampra/cmh407 10.1093/fampra/cmh407 15249526

[2] Daar AS, Singer PA, Persad DL, Pramming SK, Matthews DR, Beaglehole R, et al. Grand challenges in chronic non-communicable diseases. Nature [Internet]. 2007 [cited 2014 May 2];450:494–6. Available from: http://www.nature.com/nature/journal/v450/n7169/full/450494a.html 10.1038/450494a 18033288

[3] Global status report on noncommunicable diseases 2014: “Attaining the nine global noncommunicable diseases targets; a shared responsibility” [Internet]. World Health Organization; 2014. Available from: http://apps.who.int/iris/bitstream/10665/148114/1/9789241564854_eng.pdf?ua=1

[4] Minh HV, Ng N, Juvekar S, Razzaque A, Ashraf A, Hadi A, et al. Self-reported prevalence of chronic diseases and their relation to selected sociodemographic variables: a study in INDEPTH Asian sites, 2005. Prev Chronic Dis [Internet]. 2008 [cited 2014 May 6];5:A86. Available from: http://www.ncbi.nlm.nih.gov/pmc/articles/PMC2483549/pdf/PCD53A86.pdf PMC2483549 18558036

[5] Ahmed SM, Hadi A, Razzaque A, Ashraf A, Juvekar S, Ng N, et al. Clustering of chronic non-communicable disease risk factors among selected Asian populations: levels and determinants. Glob Health Action [Internet]. 2009 [cited 2014 Jun 2];2. Available from: http://www.ncbi.nlm.nih.gov/pmc/articles/PMC2785214/10.3402/gha.v2i0.1986 PMC2785214 20027260

[6] Pati S, Agrawal S, Swain S, Lee JT, Vellakkal S, Hussain MA, et al. Non communicable disease multimorbidity and associated health care utilization and expenditures in India: cross-sectional study. BMC Health Serv Res [Internet]. 2014 [cited 2015 Feb 18];14:451. Available from: http://bmchealthservres.biomedcentral.com/articles/10.1186/1472-6963-14-451 10.1186/1472-6963-14-451 PMC4283077 25274447

[7] Zaman MM, Bhuiyan MR, Karim MN, Zaman M, Rahman MM, Akanda AW, et al. Clustering of non-communicable diseases risk factors in Bangladeshi adults: an analysis of STEPS survey 2013. BMC Public Health [Internet]. 2015 [cited 2016 Jan 26];15. Available from: http://www.ncbi.nlm.nih.gov/pmc/articles/PMC4501055/10.1186/s12889-015-1938-4 PMC4501055 26169788

[8] World Bank, South Asia Human Development, Health Nutrition and Population. NCDs Policy Brief-Bangladesh [Internet]. Washington D.C.: Word Bank; 2011 Feb. Available from: http://siteresources.worldbank.org/SOUTHASIAEXT/Resources/223546-1296680097256/7707437-1296680114157/NCD_BD_Policy_Feb_2011.pdf

[9] Parr JD, Lindeboom W, Khanam MA, Koehlmoos TLP. Diagnosis of chronic conditions with modifiable lifestyle risk factors in selected urban and rural areas of Bangladesh and sociodemographic variability therein. BMC Health Serv Res [Internet]. 2011 [cited 2014 May 6];11:309. Available from: http://bmchealthservres.biomedcentral.com/articles/10.1186/1472-6963-11-309 10.1186/1472-6963-11-309 PMC3239323 22078128

[10] Uddin MJ, Alam N, Sarma H, Chowdhury MA, Alam DS, Niessen L. Consequences of hypertension and chronic obstructive pulmonary disease, healthcare-seeking behaviors of patients, and responses of the health system: a population-based cross-sectional study in Bangladesh. BMC Public Health [Internet]. 2014 [cited 2014 Dec 9];14:547. Available from: http://www.biomedcentral.com/1471-2458/14/547 10.1186/1471-2458-14-547 PMC4049371 24888580

[11] Mirelman AJ, Rose S, Khan JAM, Ahmed S, Peters DH, Niessen LW, et al. The relationship between non-communicable disease occurrence and poverty—evidence from demographic surveillance in Matlab, Bangladesh. Health Policy Plan [Internet]. 2016 [cited 2016 May 19];czv134. Available from: http://heapol.oxfordjournals.org/content/early/2016/02/02/heapol.czv134 10.1093/heapol/czv134 26843515

[12] Bangladesh Bureau of Statistics (BBS). District Statistics 2011: Rangpur [Internet]. Reproduction, Documentation and Publication (RDP), FA & MIS, BBS: Bangladesh Bureau of Statistics (BBS); 2013 Jun. Available from: http://www.bbs.gov.bd/WebTestApplication/userfiles/Image/District%20Statistics/Rangpur.pdf

[13] Poverty Maps of Bangladesh-2010: Technical Report [Internet]. 2014. Report No.: 90487 v2. Available from: http://www-wds.worldbank.org/external/default/WDSContentServer/WDSP/IB/2014/11/11/000442464_20141111221335/Rendered/PDF/904870v20Bangl0LIC000Sept004020140.pdf

[14] Ahmed SM, Alam BB, Anwar I, Begum T, Huque R, Khan JA, et al. Bangladesh Health System Review [Internet]. Naheed A, Hort K, editors. World Health Organisation; 2015 [cited 2016 Mar 8]. Available from: http://www.wpro.who.int/asia_pacific_observatory/hits/series/bgd_health_system_review.pdf

[15] Directorate General of Health Services, Ministry of Health and Family Welfare. Strategic plan for surveillance and prevention of non-communicable diseases in Bangladesh 2007-2010 [Internet]. Directorate General of Health Services, Ministry of Health and Family Welfare; 2007 [cited 2015 Jul 6]. Available from: https://extranet.who.int/nutrition/gina/sites/default/files/BGD%202007%20Strategic%20Plan%20NCD.pdf

[16] Directorate General of Health Services, Ministry of Health and Family Welfare. Strategic Plan for Surveillance and Prevention of Non Communicable Diseases in Bangladesh 2011-2015 [Internet]. NCD Unit, Directorate General of Health Services, Mohakhali, Dhaka.; 2011 [cited 2016 Feb 29]. Available from: http://www.searo.who.int/bangladesh/publications/strategic_plan_2011-15.pdf?ua = 1

[17] El-Saharty S, Ahsan KZ, Koehlmoos TLP, Engelgau MM. Tackling noncommunicable diseases in Bangladesh: now is the time [Internet]. Washington D.C.: The World Bank; 2013 Aug p. 1–140. Report No.: 80752. Available from: http://documents.worldbank.org/curated/en/2013/08/18165169/tackling-noncommunicable-diseases-bangladesh-now-time

[18] Short ME, Goetzel RZ, Pei X, Tabrizi MJ, Ozminkowski RJ, Gibson TB, et al. How accurate are self-reports? An analysis of self-reported healthcare utilization and absence when compared to administrative data. J Occup Environ Med Am Coll Occup Environ Med [Internet]. 2009 [cited 2016 Sep 6];51:786–96. Available from: http://www.ncbi.nlm.nih.gov/pmc/articles/PMC2745402/10.1097/JOM.0b013e3181a86671 PMC2745402 19528832

[19] Roberts RO, Bergstralh EJ, Schmidt L, Jacobsen SJ (1996). Comparison of self-reported and medical record health care utilization measures. J Clin Epidemiol..

[20] Kjellsson G, Clarke P, Gerdtham U-G. Forgetting to remember or remembering to forget: a study of the recall period length in health care survey questions. J Health Econ [Internet]. 2014 [cited 2018 Mar 10];35:34–46. Available from: http://linkinghub.elsevier.com/retrieve/pii/S0167629614000083 10.1016/j.jhealeco.2014.01.007 24595066

[21] Wang Q, Brenner S, Leppert G, Banda TH, Kalmus O, Allegri MD. Health seeking behaviour and the related household out-of-pocket expenditure for chronic non-communicable diseases in rural Malawi. Health Policy Plan [Internet]. 2015 [cited 2016 May 19];30:242–52. Available from: http://heapol.oxfordjournals.org/content/30/2/242 10.1093/heapol/czu004 24561879

[22] Ahmed SM, Tomson G, Petzold M, Kabir ZN. Socioeconomic status overrides age and gender in determining health-seeking behaviour in rural Bangladesh. Bull World Health Organ [Internet]. 2005 [cited 2014 Jun 2];83:109–17. Available from: http://www.ncbi.nlm.nih.gov/pmc/articles/PMC2623805/PMC2623805 15744403

[23] Long JS, Freese J (2001). Regression models for categorical dependent variables using Stata.

[24] Andersen RM, Davidson PL. Improving access to care in America: individual and contextual indicators. Chang US Health Care Syst Key Issues Health Serv Policy Manag [Internet]. 3rd ed. San Francisco: Jossey-Bass; 2007 [cited 2016 Mar 17]. p. 3–31. Available from: https://www.researchgate.net/profile/Ron_Andersen/publication/237675193_IMPROVING_ACCESS_TO_CARE_IN_AMERICA_Individual_and_Contextual_Indicators/links/556cd20a08aec226830548fa.pdf

[25] World Health Organization, Regional office for South-East Asia. Action plan for the prevention and control of non-communicable diseases in South-East Asia, 2013-2020 [Internet]. World Health Organization (WHO); 2013 [cited 2016 Feb 29]. Available from: http://www.searo.who.int/entity/noncommunicable_diseases/documents/sea-ncd-89(reduced).pdf?ua=1

[26] Institute for Health Metrics and Evaluation, University of Washington, Human Development Network, The World Bank. The Global Burden of Disease: Generating Evidence, Guiding Policy- South Asia Regional Edition [Internet]. Seattle, Washington, United States of America; 2013. Available from: http://www.healthdata.org/sites/default/files/files/data_for_download/2013/WorldBank_SouthAsia/IHME_GBD_WorldBank_South%20Asia_FullReport.pdf

[27] Bhuiya MMM, Khanam R, Rahman MM, Nghiem S. Microfinance, health seeking behaviour and health service of rural households: evidence from a cross-sectional study in Bangladesh. Proc 37th Annu Aust Health Econ Soc Conf [Internet]. Australian Health Economics Society; 2015 [cited 2016 Mar 18]. Available from: http://eprints.usq.edu.au/28062

[28] Sepehri A, Moshiri S, Simpson W, Sarma S. Taking account of context: how important are household characteristics in explaining adult health-seeking behaviour? The case of Vietnam. Health Policy Plan [Internet]. 2008 [cited 2016 May 19];23:397–407. Available from: http://heapol.oxfordjournals.org/content/23/6/397 10.1093/heapol/czn034 18775945

[29] Vyas S, Kumaranayake L. Constructing socio-economic status indices: how to use principal components analysis. Health Policy Plan [Internet]. 2006 [cited 2014 Jun 30];21:459–68. Available from: http://heapol.oxfordjournals.org/content/21/6/459 10.1093/heapol/czl029 17030551

[30] Ahmed SM, Hossain MA, Chowdhury MR. Informal sector providers in Bangladesh: how equipped are they to provide rational health care? Health Policy Plan [Internet]. 2009 [cited 2016 Mar 24];24:467–78. Available from: http://heapol.oxfordjournals.org/content/24/6/467 10.1093/heapol/czp037 19720721

[31] Lewis F, Butler A, Gilbert L. A unified approach to model selection using the likelihood ratio test. Methods Ecol Evol [Internet]. 2011 [cited 2016 May 19];2:155–62. Available from: http://onlinelibrary.wiley.com/doi/10.1111/j.2041-210X.2010.00063.x/full

[32] Alam D, Robinson H, Kanungo A, Hossain MD, Hassan M. Health System preparedness for responding to the growing burden of non-communicable disease-a case study of Bangladesh [Internet]. Nossal Institute for Global Health at the University of Melbourne, Australia; 2013 [cited 2016 Mar 8]. Available from: http://ni.unimelb.edu.au/__data/assets/pdf_file/0008/720656/WP25.pdf

[33] Parr J, Lindeboom W, Khanam M, Sanders J, Koehlmoos TP. Informal allopathic provider knowledge and practice regarding hypertension in urban and rural Bangladesh. PLoS ONE [Internet]. 2012 [cited 2014 Jun 13];7. Available from: http://www.ncbi.nlm.nih.gov/pmc/articles/PMC3485017/10.1371/journal.pone.0048056 PMC3485017 23133546

[34] Sarker A, Mahumud R, Sultana M, Ahmed S, Ahmed W, Khan JA. The impact of age and sex on healthcare expenditure of households in Bangladesh. SpringerPlus [Internet]. 2014 [cited 2016 Jun 27];3:435. Available from: http://springerplus.springeropen.com/articles/10.1186/2193-1801-3-435 10.1186/2193-1801-3-435 PMC4153877 25191633

[35] Pokhrel S, Snow R, Dong H, Hidayat B, Flessa S, Sauerborn R. Gender role and child health care utilization in Nepal. Health Policy [Internet]. 2005 [cited 2014 Nov 21];74:100–9. Available from: http://www.sciencedirect.com/science/article/pii/S0168851004002933 10.1016/j.healthpol.2004.12.013 16098416

[36] Grossman M. On the concept of health capital and the demand for health. J Polit Econ [Internet]. 1972 [cited 2016 Jul 18];80:223–255. Available from: http://www.jstor.org/stable/1830580

